# Acute inactivation of retromer and ESCPE-1 leads to time-resolved defects in endosomal cargo sorting

**DOI:** 10.1242/jcs.246033

**Published:** 2020-08-03

**Authors:** Ashley J. Evans, James L. Daly, Anis N. K. Anuar, Boris Simonetti, Peter J. Cullen

**Affiliations:** School of Biochemistry, Biomedical Sciences Building, University of Bristol, Bristol BS8 1TD, UK

**Keywords:** ESCPE-1, VPS35, Endosome, Retromer, Knocksideways, GLUT1, CI-MPR, SNX5

## Abstract

Human retromer, a heterotrimer of VPS26 (VPS26A or VPS26B), VPS35 and VPS29, orchestrates the endosomal retrieval of internalised cargo and promotes their cell surface recycling, a prototypical cargo being the glucose transporter GLUT1 (also known as SLC2A1). The role of retromer in the retrograde sorting of the cation-independent mannose 6-phosphate receptor (CI-MPR, also known as IGF2R) from endosomes back to the *trans*-Golgi network remains controversial. Here, by applying knocksideways technology, we develop a method for acute retromer inactivation. While retromer knocksideways in HeLa and H4 human neuroglioma cells resulted in time-resolved defects in cell surface sorting of GLUT1, we failed to observe a quantifiable defect in CI-MPR sorting. In contrast, knocksideways of the ESCPE-1 complex – a key regulator of retrograde CI-MPR sorting – revealed time-resolved defects in CI-MPR sorting. Together, these data are consistent with a comparatively limited role for retromer in ESCPE-1-mediated CI-MPR retrograde sorting, and establish a methodology for acute retromer and ESCPE-1 inactivation that will aid the time-resolved dissection of their functional roles in endosomal cargo sorting.

## Abstract (precis)

**Summary:** Retromer, a master controller of endosomal cargo sorting, is deregulated in neurodegenerative disease. Here, we develop and apply a retromer knocksideways methodology to quantify endosomal cargo sorting upon acute perturbation.

## INTRODUCTION

The endosomal pathway functions as a major intracellular hub for the sorting of numerous integral proteins, which include signalling receptors, adhesion molecules, nutrient transporters, ion channels, and their associated proteins and lipids (collectively termed ‘cargoes’) (Maxfield and McGraw, 2004; Grant and Donaldson, 2009; Cullen and Steinberg, 2018). On entering the pathway, cargoes are sorted between two fates: they are either selected for degradation within the lysosome, or retrieved from this fate and promoted for recycling to the plasma membrane and the *trans*-Golgi network (TGN) (Cullen and Steinberg, 2018). The efficient sorting of cargo is essential for normal cellular homeostasis, and defects in sorting are increasingly linked with human physiology and pathophysiology (Schreij et al., 2016; Cullen and Steinberg, 2018).

Sequence-dependent cargo sorting for retrieval and recycling is orchestrated by highly conserved multi-protein complexes that include the retromer and retriever complexes, the COMMD/CCDC22/CCDC93 (CCC) complex, and the endosomal SNX-BAR sorting complex for promoting exit-1 (ESCPE-1) complex (Seaman et al., 1998; Carlton et al., 2004; Phillips-Krawczak et al., 2015; McNally et al., 2017; Simonetti et al., 2019). These bind to sorting motifs present within the intracellular cytoplasmic domains of cargo either directly (Fjorback et al., 2012; Phillips-Krawczak et al., 2015; Bartuzi et al., 2016; Lucas et al., 2016; Kvainickas et al., 2017; Simonetti et al., 2019) or indirectly via cargo adaptors (Lauffer et al., 2010; Harterink et al., 2011; Temkin et al., 2011; Steinberg et al., 2012, 2013; Gallon et al., 2014; McNally et al., 2017). Working alongside these complexes, the endosome-associated Wiscott–Aldrich syndrome protein and SCAR homologue (WASH) complex drives the ARP2/3-mediated formation of branched F-actin networks (Derivery et al., 2009; Gomez and Billadeau, 2009). Together, cargo recognition and organisation of a localised F-actin network leads to the formation of one or more retrieval sub-domains on the cytosolic face of the endosomal membrane that provide platforms for the co-ordinated biogenesis of cargo-enriched transport carriers (Puthenveedu et al., 2010).

In higher metazoans, retromer is defined as a stable heterotrimer of VPS35, VPS29 and VPS26 (mammals express two paralogs, VPS26A and VPS26B) (Burd and Cullen, 2014). Retromer is associated to endosomes through binding to sorting nexin-3 (SNX3) (Harterink et al., 2011), RAB7-GTP (paralogs RAB7A and RAB7B) (Rojas et al., 2008; Seaman et al., 2009) and by association to cargo (Harrison et al., 2014; Lucas et al., 2016). Retromer also binds to sorting nexin-27 (SNX27), a cargo adaptor for the sequence-dependent recognition of around 400 cargo proteins that contain a specific type of C-terminal PDZ-binding motif (Temkin et al., 2011; Steinberg et al., 2013; Gallon et al., 2014; Clairfeuille et al., 2016). The principal role of retromer is therefore is to orchestrate the retrieval of hundreds of internalised cargo and to promote their recycling to the cell surface (Temkin et al., 2011; Steinberg et al., 2013). That being said, controversy remains as to the role of retromer in a distinct retrieval pathway, the retrograde endosome-to-TGN sorting of the cation-independent mannose 6-phosphate receptor (CI-MPR, also known as IGF2R) (reviewed in Seaman, 2018).

At steady state, CI-MPR is predominantly enriched at the TGN where it associates with newly synthesised hydrolase precursors (Braulke and Bonifacino, 2009). The resulting CI-MPR–hydrolase complex is transported to the endosomal pathway, where the acidified endosomal lumen induces the release of the hydrolase. While the hydrolase precursors are delivered to the lysosome, where they contribute to the degradative capacity of this organelle, the unoccupied CI-MPR is retrieved and recycled to the TGN for further rounds of hydrolase delivery. Many studies in mammalian cells are consistent with a role for retromer in CI-MPR transport (Arighi et al., 2004; Seaman, 2004, 2007; Wassmer et al., 2007; Bulankina et al., 2009; Harbour et al., 2010; Hao et al., 2013; Breusegem and Seaman, 2014; McGough et al., 2014; Cui et al., 2019). However, we, and others, have recently questioned the precise role of retromer in this pathway (Kvainickas et al., 2017; Simonetti et al., 2017). Rather, structural, biochemical and functional evidence has associated ESCPE-1 in sequence-dependent endosome-to-TGN sorting of the CI-MPR through direct recognition of a bipartite sorting motif localised within the unstructured cytosplasmic tail of this receptor (Kvainickas et al., 2017; Simonetti et al., 2017, 2019).

Part of this controversy may stem from technical variability and in particular the reliance on the generation of retromer knockdown and knockout cells (Seaman, 2018). These procedures induce the gradual loss of retromer expression over the course of hours and days, a time window that has the potential to initiate the activation of compensatory pathways that suppress phenotypes or result in variable and subtle phenotypes. Here, we have applied the ‘knocksideways’ methodology (Robinson et al., 2010) to acutely remove retromer and trap this complex on an organelle not implicated in retromer function. Using time-resolved analysis of cargo trafficking, we show that while acute retromer inactivation leads to robust defects in the endosomal recycling of the prototypical retromer cargo GLUT1 (also known as SLC2A1), we failed to detect a quantifiable perturbation in the distribution of the CI-MPR. In contrast, acute knocksideways-mediated inactivation of ESCPE-1 led to a time-resolved perturbation in CI-MPR endosome-to-TGN sorting. Our study therefore defines a method for the acute inactivation of endosomal retrieval and recycling complexes, and provides further data to support the need to reflect on the central role of retromer in the retrograde sorting of the CI-MPR.

## RESULTS

### Retromer knocksideways – design and temporal dynamics

**Fig. 1.**
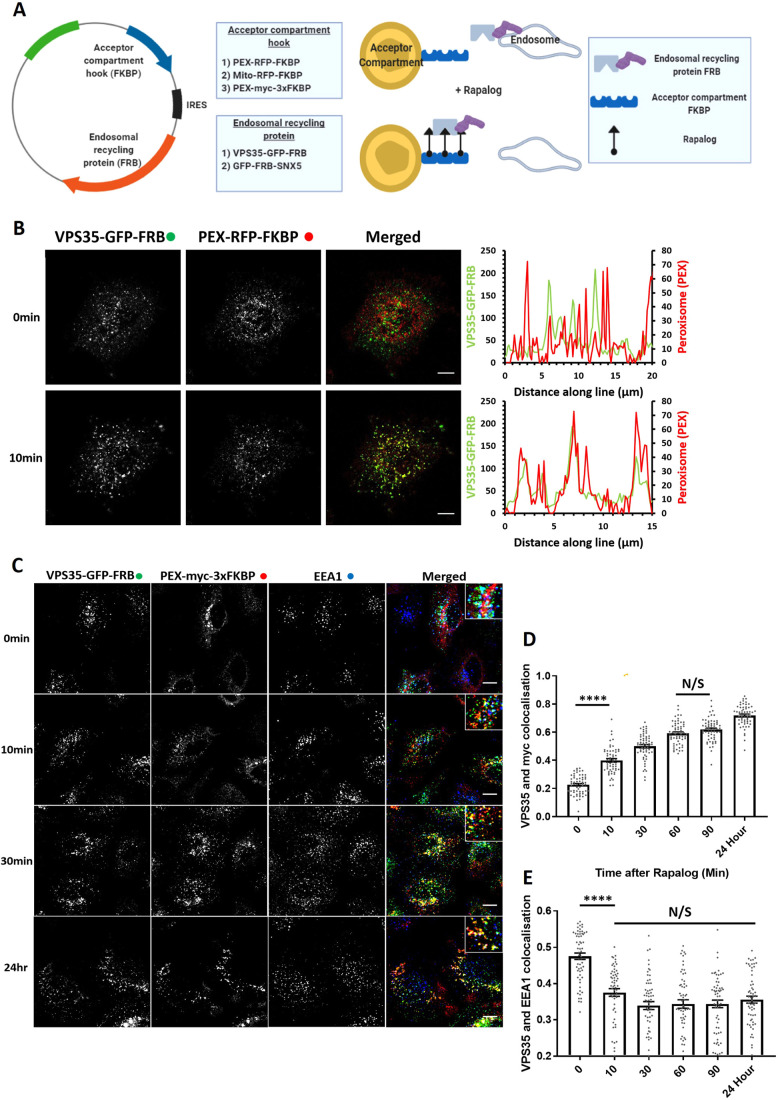
**Knocksideways can rapidly mislocalise retromer from endosomes.** (A) Schematic showing the design of the endosomal knocksideways system. (B) HeLa cells transfected with retromer knocksideways (PEX–RFP–FKBP and VPS35–GFP–FRB). Still frames are shown from a movie (Movie 1A) at either 0 min or 10 min after the addition of rapalog. Line scans were generated using ImageJ by drawing a line through peroxisome structures, and represent the colocalisation between VPS35–GFP–FRB and PEX–RFP–FKBP at each time point. The merged panel displays both channels. (C) Retromer knocksideways HeLa cells were fixed at multiple time points after the addition of rapalog. Anti-Myc and anti-EEA1 antibodies were used to label PEX–Myc–3×FKBP and early endosomes, respectively, and the merged panel displays triple colocalisation between three channels. Magnified images are displayed in the insets at the top right of the merged image. (D) Pearson’s colocalisation between VPS35–GFP–FRB and PEX–Myc–3×FKBP (peroxisomal targeting sequence) at multiple time points after the addition of rapalog. *n*_exp_=3, *n*_cell_=60 with all data points being displayed. (E) Pearson’s colocalisation between VPS35–GFP–FRB and EEA1 (early endosome marker) at multiple time points after the addition of rapalog. *n*_exp_=3, *n*=60 with all data points being displayed. ****P<0.0001; N/S, not significant (*P*>0.05) (ordinary one-way ANOVA with multiple comparisons). Error bars show the s.e.m. Scale bars: 10 µm.

To design the retromer knocksideways, we first engineered a cassette encoding the core VPS35 subunit fused through a C-terminal flexible linker to green fluorescent protein (GFP), which was itself linked to the N-terminus of rapamycin-binding (FRB) domain (resultant construct encoding VPS35–GFP–FRB, Fig. 1A). In light of evidence linking retromer to aspects of lysosomal function (e.g. Kvainickas et al., 2019), we utilised a modified version of FRB (T2098L) that enables the induction of heterodimerisation by rapalog (AP21967), a compound that has a lower affinity to endogenous mTOR than rapamycin (Clackson et al., 1998). To validate that the VPS35–GFP–FRB chimera was functional, we expressed the construct in a previously characterised VPS35-knockout HeLa cell line (Kvainickas et al., 2017). The VPS35–GFP–FRB chimera localised to cytosolic puncta that were identified as endosomes by means of colocalisation with the endosome marker EEA1 (Fig. S1A). Consistent with VPS35–GFP–FRB assembling into a functional retromer, expression of the VPS35–GFP–FRB chimera in the VPS35-knockout HeLa cells reverted the observed lysosomal missorting of GLUT1 and allowed recycling of the transporter back to the cell surface (Fig. S1B,C). The designed VPS35–GFP–FRB chimera is therefore correctly localised to endosomes and retains its function in endosomal cargo retrieval and recycling.

To engineer the acceptor compartment, we fused red fluorescent protein (RFP) to FKBP and linked this to either a mitochondrial targeting sequence [yeast Tom70p, forming Mito–RFP–FKBP (Robinson et al., 2010)] or a peroxisomal targeting sequence [PEX3 (residues 1–42), forming PEX–RFP–FKBP (Kapitein et al., 2010)]. To ensure a balanced co-expression, we cloned the genes encoding Mito–RFP–FKBP and VPS35–GFP–FRB into a bicistronic vector, and generated a corresponding bicistronic vector for PEX–RFP–FKBP and VSP35–GFP–FRB (Fig. 1A). To visualise the temporal dynamics of retromer knocksideways, we performed live imaging immediately after the application of rapalog. For both the mitochondrial and peroxisomal knocksideways systems, we observed dynamic accumulation of VPS35–GFP–FRB onto the corresponding acceptor compartment (Movies 1A and B), such that ∼10 min after induction of dimerisation there was clear colocalisation between retromer and the acceptor compartment (Fig. 1B; Fig. S1D).

Considering that retromer has been implicated in mitochondrial function (Braschi et al., 2010), we decided to focus on developing the peroxisomal acceptor compartment system; to date, peroxisomes have not been implicated in retromer biology. To increase the capacity of the acceptor compartment, we converted PEX–RFP–FKBP to PEX–Myc–3×FKBP (each FKBP separated by a flexible linker of GGSGGGSGGAP) (Fig. 1A). In transiently transfected HeLa cells, the PEX–Myc–3×FKBP chimera displayed colocalisation with the known peroxisome marker PMP70 (Fig. S1E).

In VPS35-knockout HeLa cells transiently transfected to express PEX–Myc–3×FKBP and VPS35–GFP–FRB, the addition of 100 nM of rapalog established that rerouting of VPS35–GFP–FRB from EEA1-positive endosomes to peroxisomes was achieved within 10 min and was complete by 30 min (Fig. 1C–E) – in the continued presence of rapalog the peroxisome rerouted VPS35–GFP–FRB was retained on this organelle (maximum time studied 24 h). Together, these data establish a method for the acute knocksideways of a functional VPS35–GFP–FRB construct.

### Using knocksideways to examine retromer assembly in cells

**Fig. 2.**
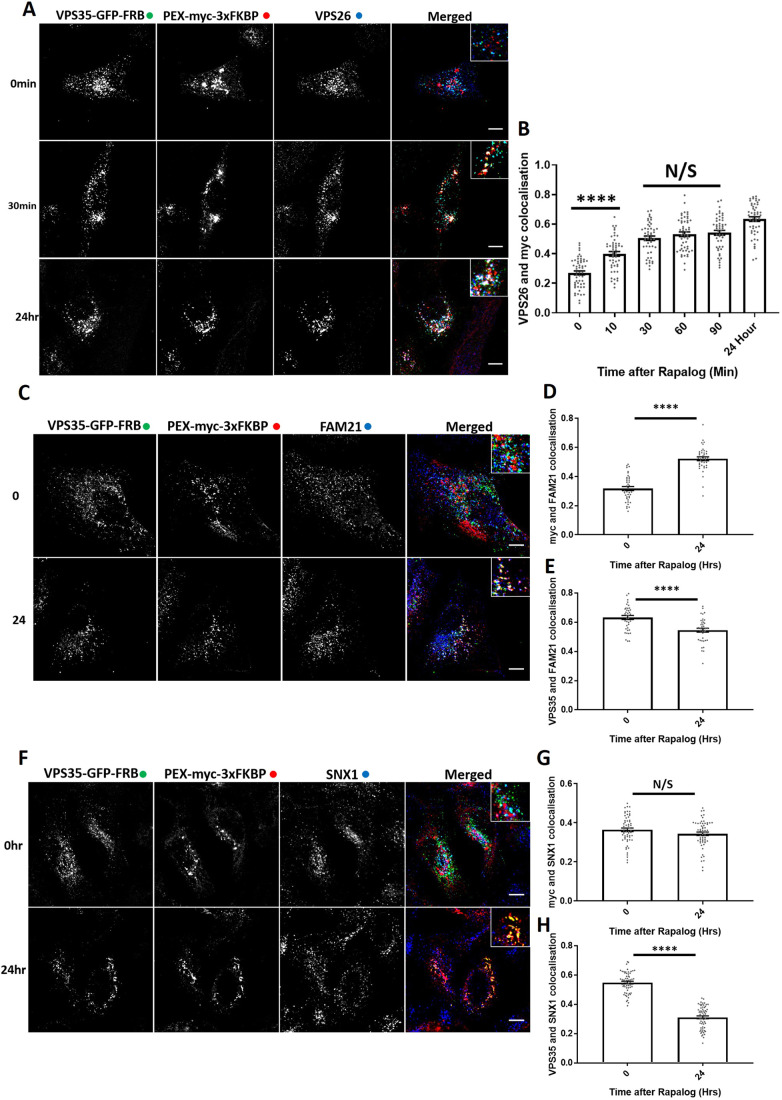
**Retromer knocksideways ‘drags’ biochemically validated interacting proteins onto peroxisomes.** (A) Retromer knocksideways HeLa cells were fixed before or at multiple time points after the addition of rapalog and labelled for Myc and VPS26. A merged panel displays all three channels combined with a magnified image (inset). (B) Pearson's colocalisation between VPS26 and Myc at multiple timepoints after the addition of rapalog. *n*_exp_=3, *n*_cell_=49-54 with all datapoints being displayed. ****P<0.0001; N/S, not significant (*P*>0.05) (ordinary one-way ANOVA with multiple comparisons). (C) Retromer knocksideways HeLa cells were fixed before or after 24 h of rapalog addition and then labelled with anti-myc and anti-FAM21 antibodies. The merged panel displays all three channels with a magnified image (inset). (D) Pearson's colocalisation between Myc and FAM21 before and after 24 h of rapalog treatment. *n*_exp_=2, *n*_cell_=40 with all data points being displayed. ****P<0.0001 (Welch's *t*-test). (E) Pearson's colocalisation between anti-VPS35 and anti-FAM21 antibodies before and after 24 h of rapalog treatment. *n*_exp_=2, *n*_cell_=40 with all data points being displayed. ****P<0.0001 (Welch's *t*-test). (F) Retromer knocksideways HeLa cells were fixed before or after 24 h of rapalog treatment and then labelled for Myc and SNX1. The merged panel displays the PEX–Myc–3×FKBP and SNX1 channels combined and with a magnified image (inset). (G) Pearson's colocalisation between Myc and SNX1 before and after the addition of rapalog for 24 h. *n*_exp_=3, *n*_cell_=60 with all datapoints being displayed. N/S, not significant (*P*>0.05) (Welch's *t*-test). (H) Pearson's colocalisation between VPS35–GFP–FRB and SNX1 before and after the addition of rapalog for 24 h. *n*_exp_=3, *n*_cell_=60 with all datapoints being displayed. ****P<0.0001 (Welch's *t*-test). Error bars show the s.e.m. Scale bars: 10 µm.

GFP–nanotrap immunoisolation is an established method for identifying protein–protein interactions, including those of the retromer complex (McGough et al., 2014; McMillan et al., 2016). Here, we used knocksideways to analyse protein–protein interactions in living cells. Consistent with the assembly of VPS35–GFP–FRB into a functional complex (Fig. S2A), analysis of the endogenous localisation of VPS26 revealed that it too was rerouted to peroxisomes with a similar kinetic profile to that observed for VPS35–GFP–FRB (the lack of a suitable antibody precluded the equivalent analysis of VPS29) (Fig. 2A,B). In addition, the major retromer accessory complex, the FAM21-containing WASH complex (Derivery et al., 2009; Gomez and Billadeau, 2009; Harbour et al., 2010; Jia et al., 2012) was also rerouted to peroxisomes upon retromer knocksideways (Fig. 2C,D; Fig. S2B,C). Supporting evidence that a sub-population of the WASH complex is associated with endosomes independently of retromer (McNally et al., 2017; MacDonald et al., 2018), a significant amount of the WASH complex was retained on endosomes even after retromer knocksideways (Fig. 2C,E; Fig. S2B,D,E). Retromer knocksideways was selective in that VPS35L, the core component of the functionally distinct retriever complex (McNally et al., 2017), retained endosome association and was not recruited to peroxisomes upon retromer knocksideways (Fig. S2F–H).

Given the selectivity of retromer knocksideways, we also decided to apply this methodology to examine the relationship between retromer and the SNX-BAR proteins that assemble to form the ESCPE-1 complex in cells (Simonetti et al., 2019). In yeast, these SNX-BAR proteins associate with the Vps26–Vps35–Vps29 heterotrimer to form the stable pentameric retromer complex (Seaman et al., 1998). In metazoans, however, retromer and ESCPE-1 appear to function independently, which is inconsistent with the formation of a long-lived and stable pentameric complex (Kvainickas et al., 2017; Simonetti et al., 2017). Indeed, we failed to observe the rerouting of endogenous SNX1, a component of the ESCPE-1 complex, onto peroxisomes after 24 h of rapalog treatment (Fig. 2F–H). These data therefore support the *in vivo* evidence that in metazoans retromer and ESCPE-1 have evolved into functionally distinct complexes (Kvainickas et al., 2017; Simonetti et al., 2017, 2019; Strutt et al., 2019). Overall, the designed VPS35 knocksideways provides a method for the acute and selective rerouting of retromer (and its functionally coupled accessory proteins) away from endosomes to neighbouring peroxisomes.

### Acute retromer knocksideways leads to a time-resolved GLUT1 sorting defect

**Fig. 3.**
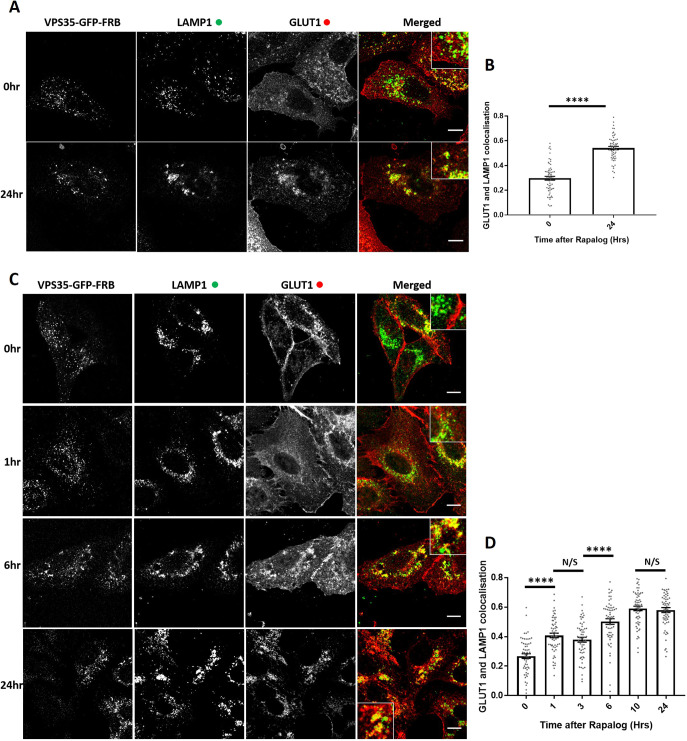
**Retromer knocksideways results in the rapid functional inactivation of retromer and the temporal resolution of the accumulation of retromer-depleted phenotypes.** (A) Retromer knocksideways HeLa cells were fixed before or after 24 h of rapalog addition. Anti-LAMP1 and anti-GLUT1 were then used to label the late endosome/lysosome and retromer cargo, respectively. The merge panel displays both the LAMP1 (green) and the GLUT1 (red) channels with a magnified image (inset). (B) Pearson's colocalisation between GLUT1 and LAMP1 before and after 24 h of rapalog treatment. *n*_exp_=3, *n*_cell_=60 with all data points being displayed. ****P<0.0001 (Welch's *t*-test). (C) Retromer knocksideways HeLa cells were fixed before and after the indicated time of rapalog treatment. Anti-LAMP1 and anti-GLUT1 were then used to label late endosome/lysosome and retromer cargo, respectively. The merge panels display both the LAMP1 and GLUT1 labelling with a magnified image (inset). (D) Pearson's colocalisation between GLUT1 and LAMP1 before and at multiple time points after the addition of rapalog. *n*_exp_=3, *n*_cell_=60 with all data points being displayed. ****P<0.0001; N/S, not significant (*P*>0.05) (ordinary one-way ANOVA with multiple comparisons). Error bars show the s.e.m. Scale bars: 10 µm.

Retromer and retromer-associated cargo adaptors have been shown to control the endosomal retrieval and recycling of numerous cell surface proteins including the glucose transporter GLUT1 (Steinberg et al., 2013). To define the functional consequence of retromer knocksideways, we examined the steady-state distribution of GLUT1 in VPS35-knockout HeLa cells rescued by expression of the VPS35–GFP–FRB knocksideways construct. Following the addition of rapalog for 24 h, fixed cell confocal imaging revealed a GLUT1 missorting phenotype, defined by the steady-state loss of GLUT1 at the cell surface and the enrichment of GLUT1 with LAMP1-positive late endosomes/lysosomes (Fig. 3A,B). To time-resolve the appearance of the GLUT1 trafficking phenotype, we fixed cells at various points following rapalog addition. Quantification established that a statistically significant GLUT1 missorting phenotype began to emerge after 1–3 h of retromer knocksideways and reached a maximum penetrance after 10 h (Fig. 3C,D). The difference between the time scales of retromer knocksideways (Fig. 1C–E) compared with the appearance of the GLUT1 missorting phenotype is entirely consistent with the known rate of GLUT1 lysosomal-mediated degradation observed upon retromer suppression and reflects the relatively slow rate of endocytosis of this transporter (Steinberg et al., 2013).

The missorting of GLUT1 upon retromer knocksideways was not the result of a global effect on endosomal sorting, as the endosomal retrieval and recycling of the retriever-dependent cargo α_5_β_1_-integrin (McNally et al., 2017) was not affected upon retromer knocksideways (Fig. S3A,B) – consistent with the lack of effect of retromer knocksideways on the endosomal association of the retriever complex (Fig. S2F–H). Moreover, the development of the GLUT1 missorting did not stem from the recruiting of ‘foreign’ proteins to peroxisomes as retromer knocksideways performed in wild-type HeLa cells, which retain expression of endogenous VPS35 that is not subject to knocksideways, did not elicit the development of a GLUT1 missorting phenotype (Fig. S3C,D). Together, these data support that it is the specific removal and inactivation of retromer that causes the time-resolved development of the observed GLUT1 missorting phenotype.

### Retromer-independent CI-MPR retrograde trafficking

**Fig. 4.**
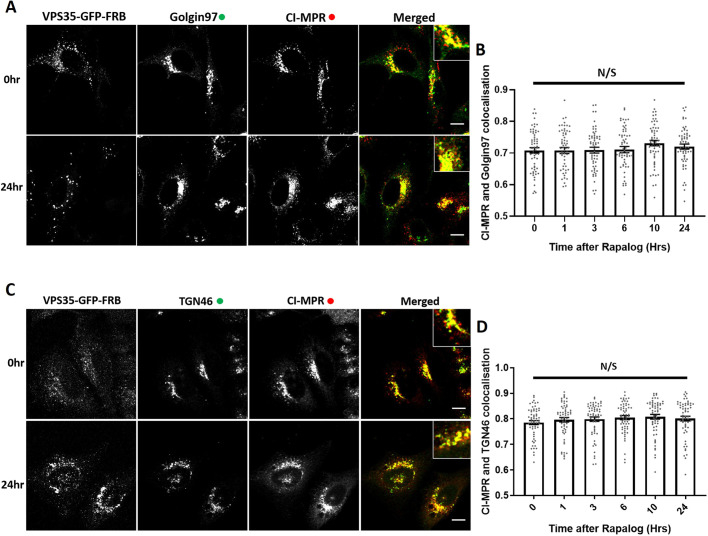
**Knocksideways indicates no visible role for retromer in SNX-BAR mediated retrograde transport of CI-MPR.** (A) Retromer knocksideways HeLa cells were fixed before or at multiple time points after the addition of rapalog and labelled for Golgin97 and CI-MPR. A merged panel displays both the anti-Golgin97 and anti-CI-MPR channels with a magnified image (inset). (B) Pearson's colocalisation between CI-MPR and Golgin97 at multiple timepoints after the addition of rapalog. *n*_exp_=3, *n*_cell_=60 with all datapoints being displayed. (C) Retromer knocksideways HeLa cells were fixed before or at multiple time points after the addition of rapalog and labelled for TGN46 and CI-MPR. A merged panel displays both the TGN46 and CI-MPR channels with a magnified image (inset). (D) Pearson's colocalisation between CI-MPR and TGN46 at multiple timepoints after the addition of rapalog. *n*_exp_=3, *n*_cell_=60 with all datapoints being displayed. N/S, not significant (*P*>0.05) (ordinary one-way ANOVA with multiple comparisons). Error bars show the s.e.m. Scale bars: 10 µm.

Next, we investigated the role of retromer in the retrograde trafficking of CI-MPR from endosomes to the TGN (Arighi et al., 2004; Seaman, 2004; Kvainickas et al., 2017; Simonetti et al., 2017; Cui et al., 2019). In VPS35-knockout HeLa cells rescued through expression of VPS35–GFP–FRB, the CI-MPR is chiefly localised to the perinuclear TGN, as defined through colocalisation with TGN markers Golgin97 and TGN46 (also known as GOLGA1 and TGOLN2, respectively) (Fig. 4A,B). After the addition of rapalog and initiation of retromer knocksideways, we failed to observe a quantifiable alteration in the steady-state distribution of the CI-MPR (Fig. 4A–D) over a time frame where the endosomal missorting of internalised GLUT1 was readily observed (Fig. 3C,D). Given that the endosome-to-TGN transport of the CI-MPR is considered to occur over a period of ∼20 to 30 min (Seaman, 2004), the acute perturbation of retromer function does not appear to lead to a detectable defect in the endosomal sorting of the CI-MPR (Kvainickas et al., 2017; Simonetti et al., 2017).

### Knocksideways of ESCPE-1 leads to a time-resolved defect in CI-MPR sorting

**Fig. 5.**
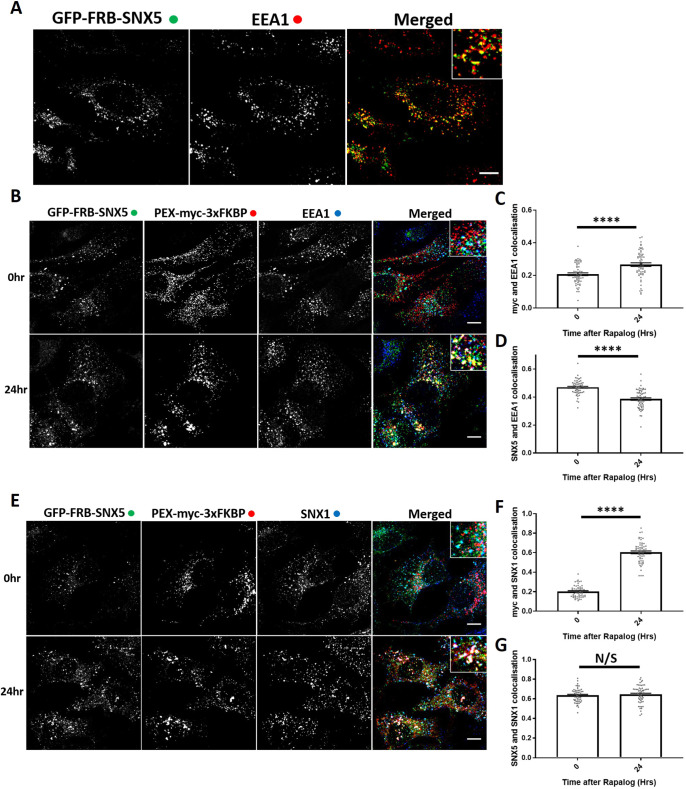
**ESCPE-1 knocksideways results in the dragging of a small population of endosomes and peroxisomes together while still removing ESCPE-1 from endosomes.** (A) SNX-BAR knocksideways cells were fixed and then labelled with anti-EEA1 antibody. Both the GFP–FRB–SNX5 and EEA1 channels are shown in the merged panel with a magnified image (inset). (B) ESCPE-1 knocksideways HeLa cells were fixed before and after the addition of rapalog for 24 h and then labelled for Myc and EEA1. The merged panel shows all three channels with a magnified image (inset). (C) Pearson's colocalisation between Myc and EEA1 before and after 24 h of rapalog treatment. *n*_exp_=3, *n*_cell_=60 with all data points being displayed. (D) Pearson's colocalisation between GFP–FRB–SNX5 and EEA1 before and after 24 h of rapalog treatment. *n*_exp_=3, *n*_cell_=60 with all data points being displayed. (E) ESCPE-1 knocksideways HeLa cells were fixed before and after the addition of rapalog for 24 h and then labelled for Myc and SNX1. The merged panel shows all three channels with a magnified image (inset). (F) Pearson's colocalisation between Myc and SNX1 before and after 24 h of rapalog treatment. *n*_exp_=3, *n*_cell_=53-60 with all data points being displayed. (G) Pearson's colocalisation between GFP–FRB–SNX5 and SNX1 before and after 24 h of rapalog treatment. *n*_exp_=3, *n*_cell_=53–60 with all data points being displayed. ****P<0.0001; N/S, not significant (*P*>0.05) (Welch's *t*-test). Error bars show the s.e.m. Scale bars: 10 µm.

The ESCPE-1 complex regulates sequence-dependent endosome-to-TGN transport of the CI-MPR (Simonetti et al., 2019). ESCPE-1 comprises a heterodimer of SNX1 or SNX2 (these proteins are functionally redundant) associated with either SNX5 or SNX6, which are also functionally redundant (Wassmer et al., 2007). Of these proteins, it is the PX domains of SNX5 and SNX6 that directly bind to the ΦxΩxΦ(x)nΦ sorting motif (where Φ represents hydrophobic amino acids) in CI-MPR to mediate endosome-to-TGN transport (Simonetti et al., 2019). To provide a positive control for the lack of detectable effect of retromer knocksideways on CI-MPR trafficking, we therefore constructed a bicistronic vector encoding PEX–Myc–3×FKBP and GFP–FRB–SNX5 (Fig. 1A). When expressed in HeLa cells, GFP–FRB–SNX5 localised to endosomes as defined by colocalisation with EEA1 (Fig. 5A). Interestingly, after rapalog addition, we observed a slight recruitment of EEA1 to the peroxisomal hook, indicating a movement of the endosomal compartment to the peroxisomal compartment (Fig. 5B,C). However, this endosomal ‘dragging’ was not complete, as there was still a loss of colocalisation between GFP–FRB–SNX5 and EEA1 (Fig. 5B,D). In GFP–FRB–SNX5 knocksideways cells, endogenous SNX1 was recruited to peroxisomes after rapalog treatment with no loss of colocalisation between SNX5 and SNX1, indicating a recruitment of the functional ESCPE-1 complex (Fig. 5E–G).

**Fig. 6.**
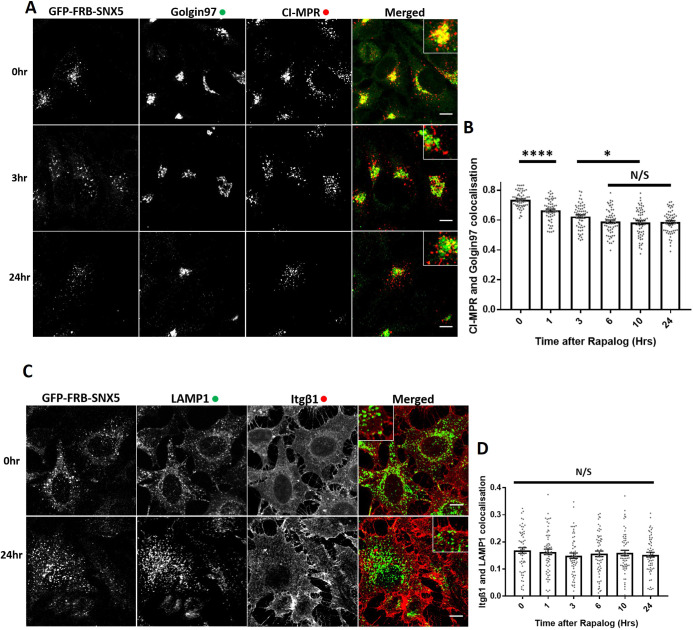
**ESCPE-1 knocksideways inactivates ESCPE-1 and results in a temporally resolved CI-MPR redistribution away from the TGN.** (A) SNX-BAR knocksideways HeLa cells were fixed before or at multiple time points after the addition of rapalog and then labelled for Golgin97 and CI-MPR. The merged panel shows both the anti-Golgin97 and anti-CI-MPR channels with a magnified image (inset). (B) Pearson's colocalisation between Golgin97 and CI-MPR before or at multiple time points after the addition of rapalog. *n*_exp_=3, *n*_cell_=60 with all data points being displayed. (C) SNX-BAR knocksideways HeLa cells were fixed before or at multiple time points after the addition of rapalog and then labelled for LAMP1 and Itgβ1. The merged panel shows both the LAMP1 and Itgβ1 channels with a zoom panel. (D) Pearson's colocalisation between LAMP1 and Itgβ1 before or at multiple time points after the addition of rapalog. *n*_exp_=3, *n*_cell_=60 with all data points being displayed. *P<0.05; ****P<0.0001; N/S, not significant (*P*>0.05) (ordinary one-way ANOVA with multiple comparisons). Error bars show the s.e.m. Scale bars: 10 µm.

Next, we used the GFP–FRB–SNX5 knocksideways system to time-resolve CI-MPR endosome-to-TGN trafficking. Expression of the GFP–FRB–SNX5 chimera in a previously isolated and characterised SNX5/SNX6 double-knockout HeLa cell line (Simonetti et al., 2017) reverted the observed missorting of the CI-MPR and allowed the receptor to return to its normal steady-state localisation (Fig. S4A,B). Consistent with the role of SNX5 in the ESCPE-1-mediated endosome-to-TGN transport of the CI-MPR (Kvainickas et al., 2017; Simonetti et al., 2017, 2019), SNX5 knocksideways in SNX5/SNX6 double-knockout HeLa cells led to the time-resolved appearance of a CI-MPR missorting phenotype as defined by a reduced enrichment of the CI-MPR at the Golgin97 or TGN46-labelled TGN with a maximum penetrance at 6 h (Fig. 6A,B; Fig. S4C,D). There was no observed defect in α_5_β_1_-integrin recycling during GFP–FRB–SNX5 knocksideways, indicating the selective nature of this procedure (Fig. 6C,D). Together, these data establish that acute perturbation of the ESCPE-1 complex leads to a missorting of CI-MPR.

### Establishing knocksideways in a human H4 neuroglioma cell line

**Fig. 7.**
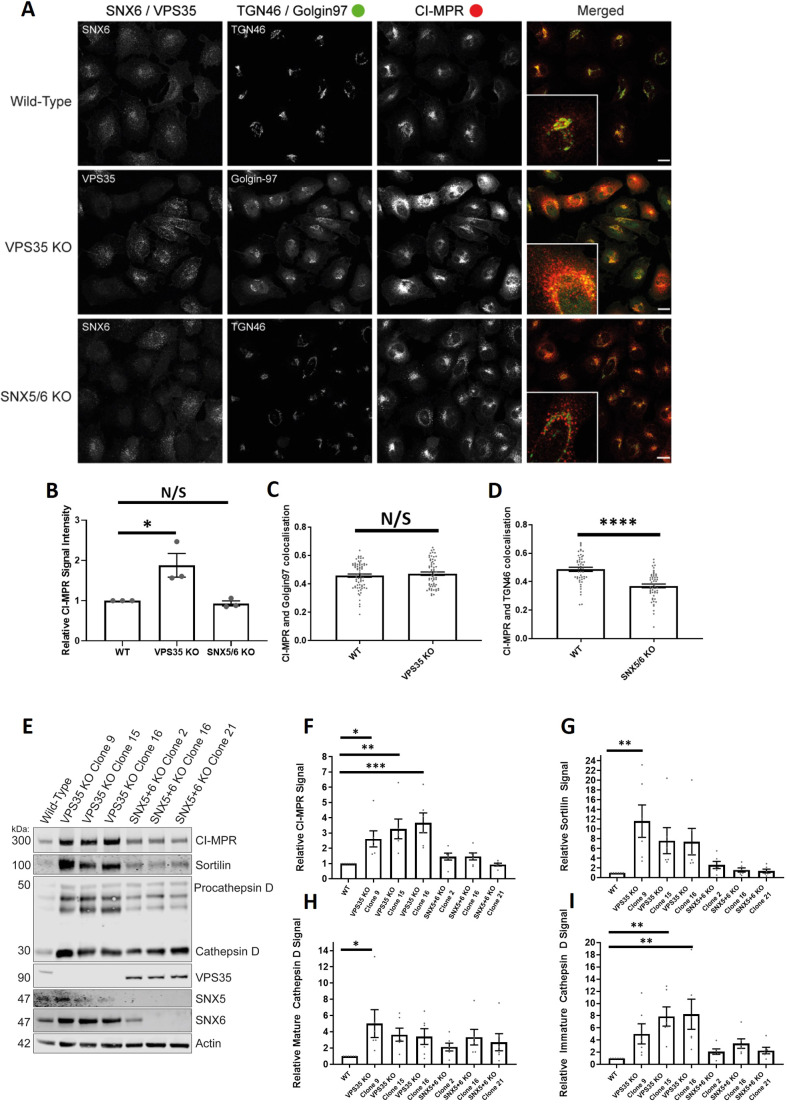
**VPS35-knockout H4 neuroglioma cells display an upregulation of lysosomal hydrolases and lysosomal hydrolase receptors.** (A) VPS35 and SNX5/SNX6 dual-knockout mixed population H4 neuroglioma cells were generated and then fixed. Cells were stained with either anti-VPS35 or anti-SNX6 antibodies to confirm which cells were knocked out in the mixed population. Cells were also co-stained with both anti-CI-MPR and either anti-TGN46 (SNX5/SNX6 dual knockout) or anti-Golgin97 (VPS35 knockout) antibodies. The merged panel displays both the CI-MPR and TGN46 or Golgin97 channels. Scale bars: 20 µm. (B) Normalised values for relative CI-MPR signal intensity between conditions. *n*_exp_=3, *n*_cell_=44–68 with average value data being shown for each experiment. *P*<0.05; N/S, not significant (*P*>0.05) (Student's *t*-test). (C) Pearson's colocalisation between CI-MPR and Golgin97 in wild-type and VPS35-knockout cells. *n*_exp_=3, *n*_cell_=64–69 with all data points being displayed. N/S, not significant (*P*>0.05) (Welch's *t*-test). (D) Pearson's colocalisation between CI-MPR and TGN46 in wild-type and SNX5/SNX6 knockout cells. *n*_exp_=3, *n*_cell_=49–53 with all data points being displayed. ****P<0.0001 (Welch's *t*-test). (E) Representative western blot analysis of wild-type and VPS35-knockout or SNX5/SNX6 knockout clonal cell lines using anti-CI-MPR, anti-sortilin, anti-cathepsin D, anti-VPS35, anti-SNX5, anti-SNX6 amd anti-actin antibodies. (F–I) Relative (actin) measured signals for wild-type and VPS35-knockout or SNX5/SNX6 dual-knockout clonal cell lines for CI-MPR, sortilin, mature cathepsin D and immature cathepsin D. *n*=7. *P<0.05; **P<0.01; ***P<0.001 (ordinary one-way ANOVA with multiple comparisons). Error bars show the s.e.m.

Our study of endosomal cargo sorting associated with depletion or knocksideways of sorting machinery has so far been limited to a single non-neuronal cell type. To extend these observations, we therefore turned to H4 neuroglioma cells and generated both retromer knockout (targeting VPS35) and ESCPE-1-knockout cells (dual targeting of SNX5 and SNX6). Interestingly, confocal imaging of the retromer-knockout cells revealed an enhanced intensity in the staining of endogenous CI-MPR that was not observed in the ESCPE-1-knockout cells (Fig. 7A,B). Despite the increase in the CI-MPR signal intensity, retromer-knockout cells did not display a significant change in the quantified Pearson's correlation coefficient between CI-MPR and Golgin97 (Fig. 7A,C). In contrast, the ESCPE-1 knockout H4 neuroglioma cells displayed a clear redistribution of CI-MPR to peripheral dispersed puncta (Fig. 7A,D; Fig. S5A).

To extend these data, we isolated individual clonal retromer and ESCPE-1-knockout H4 cell lines. Biochemical analysis of three independent clonal lines revealed that retromer knockout resulted in a pronounced upregulation of CI-MPR protein levels (Fig. S5B). Moreover, the abundance of another lysosomal hydrolase receptor, sortilin, was also increased across all three independent lines, as was the immature and mature forms of the lysosomal hydrolase cathepsin D (Fig. 7E–I). These increases in protein levels were not observed across three independent ESCPE-1-knockout H4 cell lines (Fig. 7E–I).

**Fig. 8.**
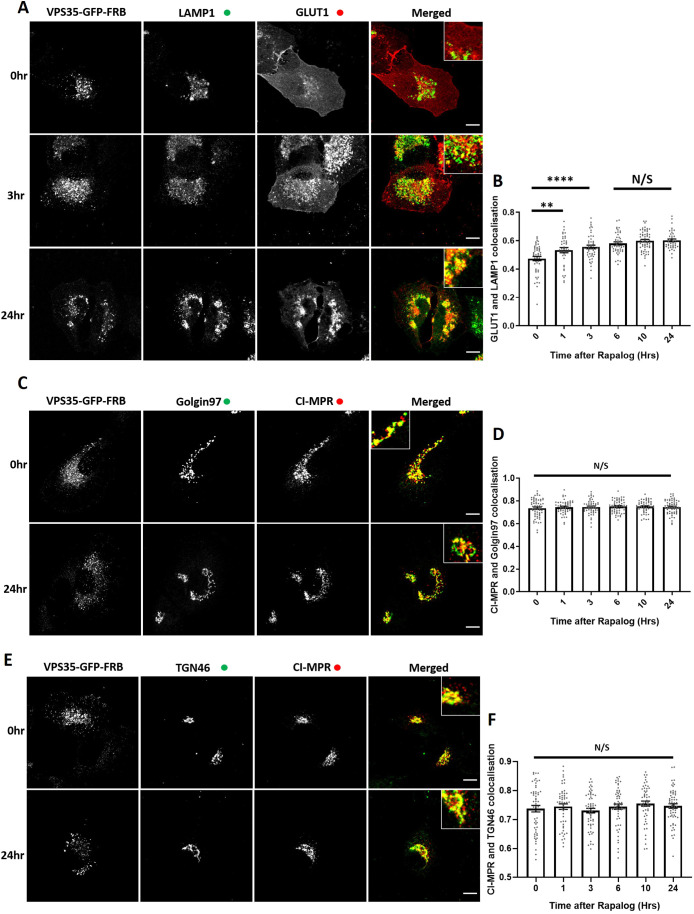
**VPS35 knocksideways in H4 neuroglioma cells confirms a role for retromer in recycling of GLUT1 but no visible role for retromer in the retrograde CI-MPR trafficking.** (A) Retromer knocksideways H4 neuroglioma cells were fixed before or at multiple time points after the addition of rapalog. Antibodies towards anti-LAMP1 and anti-GLUT1 were then used to label late endosome/lysosome and retromer cargo, respectively. The merge panels display both the LAMP1 and GLUT1 labelling with a magnified image (inset). (B) Pearson's colocalisation between GLUT1 and LAMP1 before or at multiple time points after the addition of rapalog. *n*_exp_=3, *n*_cell_=37–58 with all data points being displayed. (C) Retromer knocksideways H4 neuroglioma cells were fixed before or or at multiple time points after the addition of rapalog and labelled with anti-Golgin97 and anti-CI-MPR antibodies. A merged panel displays both the Golgin97 and CI-MPR channels with a magnified image (inset). (D) Pearson's colocalisation between CI-MPR and Golgin97 at multiple timepoints after the addition of rapalog. *n*_exp_=3, *n*_cell_=52–60 with all data points being displayed. (E) Retromer knocksideways H4 neuroblastoma cells were fixed before or at multiple time points after the addition of rapalog and labelled with anti-TGN46 and anti-CI-MPR antibodies. A merged panel displays both the TGN46 and CI-MPR channels. (F) Pearson's colocalisation between CI-MPR and TGN46 at multiple time points after the addition of rapalog. *n*_exp_=3, *n*_cell_=52–56 with all datapoints being displayed. **P<0.01; ****P<0.0001; N/S, not significant (*P*>0.05) (ordinary one-way ANOVA with multiple comparisons). Error bars show the s.e.m. Scale bars: 10 µm.

To examine whether the increased protein abundance of CI-MPR, sortilin and cathepsin D arose from a retromer-dependent trafficking defect or reflected a longer-term compensatory mechanism, we established the acute VPS35 knocksideways methodology in H4 cells. Expression of VPS35–GFP–FRB rescued the GLUT1 missorting phenotype in retromer-knockout cells. Initiation of VPS35 knocksideways resulted in a time-resolved missorting and accumulation of GLUT1 to LAMP1-positive late endosomes and lysosomes (Fig. 8A,B) confirming an acute perturbation in retromer function (Fig. 3C,D). In a parallel time-resolved retromer knocksideways experiments, we failed to detect a significant redistribution of CI-MPR away from TGN markers Golgin97 and TGN46 (Fig. 8C–F). These data in H4 cells therefore corroborates our observation in HeLa cells, and does not appear to explain the increased protein abundance of CI-MPR, sortilin and cathepsin D.

## DISCUSSION

Here, we have developed and applied knocksideways to acutely inactivate retromer and the ESCPE-1 complex (Fig. 1A). Previously developed to inactivate the AP1 and AP2 clathrin adaptors (Robinson et al., 2010; Hirst et al., 2012), this approach provides a method to acutely perturb the function of sorting complexes in a time frame that better aligns with the dynamic nature of endosomal membrane trafficking. By visualising the sorting of endogenous GLUT1 and CI-MPR, our data provide insight into the temporal dynamics of endosomal cargo sorting and support the established role of retromer in cell surface recycling (Temkin et al., 2011; Steinberg et al., 2013).

In applying knocksideways, we have established that retromer and ESCPE-1 can be specifically and rapidly inactivated, leading to the time-resolved development of selective cargo sorting defects through the endosomal pathway. Interestingly, in examining CI-MPR phenotypes in H4 neuroglioma cells, we observed a clear distinction between acute retromer knocksideways and the long-term effects of retromer knockout. Only in the latter did we observe an increase in the steady-state expression of CI-MPR, sortilin and cathepsin D (Fig. S5C). In part, this phenotype may reflect the role of retromer as a master regulator (Jimenez-Orgaz et al., 2018) of the activity state of endosomal RAB7 through binding to the RAB7 GAP TBC1D5 (Seaman et al., 2009, 2018; Jimenez-Orgaz et al., 2018; Kvainickas et al., 2019). In the absence of retromer, RAB7 loses its dynamic organisation on endosomes and lysosomes and becomes hyperactivated and immobile on lysosomes (Jimenez-Orgaz et al., 2018; Seaman et al., 2018). This leads to impaired mTORC1 activity and the induction of autophagy, and the appearance of swollen and evenly dispersed lysosomes (Jimenez-Orgaz et al., 2018; Cui et al., 2019; Curnock et al., 2019; Kvainickas et al., 2019). Moreover, retromer-knockout cells display activation of the TFEB transcription factors (Curnock et al., 2019), master regulators of cellular nutrient sensing and energy metabolism (Sardiello et al., 2009; Settembre et al., 2011). Thus, besides its role in cargo retrieval and recycling within the endosomal pathway (Steinberg et al., 2013), retromer has emerged as a master regulator of RAB7 in nutrient sensing and signalling (Jimenez-Orgaz et al., 2018; Curnock et al., 2019; Kvainickas et al., 2019). The observed upregulation of CI-MPR, sortilin and cathepsin D expression in H4 neuroglioma cells therefore likely reflects this regulatory role, through a complex compensation in lysosomal function induced by long-term retromer knockout.

In yeast, retromer is a pentameric assembly (Seaman et al., 1998). An increasing body of biochemical, cellular and *in vivo* functional data are consistent with the equivalent metazoan assembly having evolved into two functionally distinct complexes, the retromer (VPS26–VPS35–VPS29) and the ESCPE-1 (SNX1/SNX2 and SNX5/SNX6) complexes (Norwood et al., 2011; Swarbrick et al., 2011; Kvainickas et al., 2017; Simonetti et al., 2017, 2019; Strutt et al., 2019). In utilising knocksideways as an interaction assay in living cells, we have provided further supporting evidence of the distinct nature of the retromer and ESCPE-1 complexes. Specifically, acute knocksideways of the core VPS35 retromer component results in the equivalent time-resolved knocksideways of the endogenous population of VPS26 but has no detectable effect on the endosomal localisation of ESCPE-1. This technically distinct approach therefore provides further data to support the diversification of retromer and ESCPE-1 into two functionally distinct sorting complexes.

The development of retromer knocksideways has added to our understanding of the endosomal association of the actin polymerising WASH complex. Direct binding of FAM21 to VPS35 is a major mechanism for the retromer-dependent association of the WASH complex to endosomes (Gomez and Billadeau, 2009; Harbour et al., 2010; Jia et al., 2012). That said, increasing evidence suggests that a subpopulation of the WASH complex is associated to endosomes independently of retromer (McNally et al., 2017; Kvainickas et al., 2017; Simonetti et al., 2017; MacDonald et al., 2018). Consistent with these data, acute knocksideways of retromer induces a redistribution of a major proportion of endogenous WASH, but a significant subpopulation retains an endosomal association.

In summary, by applying knocksideways, we have acutely inactivated retromer and ESCPE-1 and, through quantification of the resulting temporal development of cargo-sorting defects, provided clarification of the role of these complexes in the sorting of CI-MPR and GLUT1 (Fig. S5C). While not excluding a role for retromer in the known complexities of CI-MPR sorting (Seaman, 2018), our time-resolved analysis establishes that the ESCPE-1 complex is the primary mediator of sequence-dependent endosome-to-TGN sorting of this receptor.

## MATERIALS AND METHODS

### Antibodies

Antibodies used in this study are as follows: SNX1 [clone 51; 611482; BD Bioscience; immunofluorescence (IF) 1:200], GLUT1 (ab40084; Abcam; IF 1:200), Golgin97 (clone CDF4; A-21270; Thermo Fisher Scientific; IF 1:400), VPS26 (ab23892; Abcam; IF 1:200), VPS35 (ab10099; Abcam; IF 1:200), VPS35 [ab97545; Abcam; IF 1:200), VPS35 [ab157220; Abcam; western blotting (WB) 1:1000], VPS29 (ab98929; Abcam; WB 1:200), FAM21 (gift from Daniel D. Billadeau, Mayo Clinic, Rochester, MN; IF 1:400), EEA1 (N-19; sc-6415; Santa Cruz Biotechnology; IF 1:200), TGN46 (AHP500G; Bio-Rad Laboratories; IF 1:200), anti-Myc (gift from Harry Mellor, The University of Bristol, UK; IF 1:500), LAMP1 (DSHB Hybridoma Product; H4A3; deposited to the DSHB by August, J.T./Hildreth, J.E.K.; IF 1:500), LAMP1 (ab24170; Abcam; IF 1:200), mouse EEA1 (610457; BD Bioscience; IF 1:200), CI-MPR (ab124767; Abcam; WB 1:1000, IF 1:200), Itgβ1 (TS2/16; IF 1:200), SNX6 (Clone D-5; 365965; Santa Cruz Biotechnology; WB 1:500), PMP70 (PA1-650; Invitrogen; IF 1:200), WASH1 (gift from Daniel D. Billadeau; IF 1:400), C16orf62 (PA5-28553; Pierce; IF 1:200), sortilin (ab16640; Abcam; WB 1:1000), cathepsin D (21327-1-AP, Proteintech; WB 1:1000), SNX5 (ab180520; Abcam; WB 1:500) and β-actin (A1978; Sigma-Aldrich; WB 1:2000).

### Plasmids

A pIRESneo3 vector was adapted to generate the bicistronic knocksideways system. First, VPS35–GFP and FRB (PCR from the Kinesin-FRB template (gift from Lukas Kapetein, Utrecht University, Netherlands) were PCR overlapped together inserting a XhoI site between VPS35–GRP and FRB and then ligated downstream of the IRES component between the SmaI and PacI sites. PEX–RFP–FKBP was amplified from the template (gift from Lukas Kapetein) and ligated into the MCS downstream of the CMV promoter in pIRESNEO3 using EcoRV and NotI. The mitochondrial targeting sequence (gift from Scottie Robinson CIMR, UK) was inserted in place of the PEX targeting sequence using the EcoRV and AgeI restriction sites. To create GFP–FRB–SNX5, first, GFP–FRB was amplified and inserted between the SmaI and PacI sites to generate a new FseI site upstream of the PacI restriction site. The new FseI and PacI site was used to insert SNX5. The PEX–RFP–FKBP was converted to PEX–Myc–3×FKBP by PCR of Myc–FKBP and inserted between the AgeI and NotI sites to generate PEX–Myc–FKBP. The AscI site (upstream of FKBP in the PEX–RFP–FKBP) was used to sequential insert two FKBP cassettes using a MluI-AscI insertion (MluI compatible with AscI but destroying the AscI site allowing the second insertion). CRISPR Cas9 plasmids were obtained from Addgene (#62988, pSpCas9(BB)-2A-Puro PX459 V2.0).

### Cell culture and DNA transfection

HeLa (American Type Culture Association) and H4 neuroglioma cells (we thank Dr Helen Scott and Professor James Uney for providing this cell line) were cultured in DMEM (Sigma) supplemented with 10% (v/v) FCS (Sigma) and grown using standard conditions. Lipofectamine LTX was used in DNA transfections. For each six well dish, 2 µg of DNA was mixed with 4 µl of the LTX supplement into 100 µl of Opti-Mem (Thermo Fisher). In another incubation, 100 µl of Opti-Mem was mixed with 8 µl of Lipofectamine LTX. After a 5-min incubation, the two 100 µl Opti-Mem mixes were combined and incubated for a further 20 min. The 200 µl mix was then added dropwise onto 60–80% confluent HeLa cells and transfected cells were left for 48 h for DNA expression. VPS35-knockout HeLa cells and SNX5/SNX6 double knockout was generated as previously described and cultured as stated above for wild-type HeLa cells (Simonetti et al., 2017).

### Generation of H4 clonal cells

H4 cells were seeded the day prior to transfection, then transiently transfected with CRISPR plasmids encoding the Cas9 enzyme, a puromycin-resistance marker and specific gRNA guides against VPS35, SNX5 or SNX6 (Kvainickas et al., 2017; Simonetti et al., 2017) using FuGENE^®^ 6 (Promega). The day after transfection, cells are incubated with 1 μg/ml puromycin for 24 h to select for knockout cells. Following puromycin selection, H4 cells were seeded into a 96-well plate at a density of 1 cell per well in 200 μl Iscove's modified Dulbecco's medium (Thermo Fisher). Clones were grown to confluency then expanded and screened for successful knockout deletion by western blotting.

### GFP trap and western blot analysis

Cells were lysed in GFP trap buffer (50 mM Tris-HCl, 0.5% NP-40 and Roche protease inhibitor cocktail) and the lysate was added to pre-equilibrated GFP trap beads (ChromoTek). Beads were washed three times in the GFP trap buffer and then lysates were diluted in 2× sample buffer and boiled for 10 min. Proteins were resolved on a NuPAGE 4-12% gels (Invitrogen) and transferred onto polyvinylidene fluoride membrane (EMD Millipore). Once transferred membranes were blocked in TBS 5% milk and the primary antibody (see antibody section) was diluted in Tris-buffered saline (TBS) with 0.1% (v/v) Tween-20 (TBS-T) and 5% (w/v) milk and incubated with the membrane for 1 h at room temperature or overnight at 4°C. Membranes were washed three times in TBS-T. Secondary antibodies (see antibody section) were diluted into TBS-T with 5% milk and 0.01% SDS and incubated with the washed membrane for 1 h at room temperature. TBS-T was used to wash the membrane (three times) prior to quantitative imaging using an Odyssey scanning system (LI-COR Biosciences). Analysis was performed on Image Studio Lite (LI-COR Biosciences).

### Knocksideways

pIRESneo3 bicistronic vectors encoding the knocksideways peroxisomal/mitochondrial acceptor compartment and either VPS35–GFP–FRB or GFP–FRB–SNX5 were transfected into cells. Either 0.1% (v/v) ethanol vehicle or rapalog (Takara, Cat. #635056, 100 nM) was added at the 0 timepoint and cells were cultured for a further 24 h. The following day rapalog was added for a further period as indicated, and then cells were fixed and stained.

### Immunofluorescence staining

Cells were washed once in PBS before fixation for 8 min in 4% PFA (16% PFA stock diluted in PBS). Three washes in PBS were performed, and then a 5-min incubation with PBS 100 mM glycine was used to quench the PFA. After three more PBS washes, cells were left in PBS overnight. Cells were incubated with PBS plus 3% BSA and 0.1% Triton X-100 for 10 min and then with PBS plus 3% BSA for a further 10 min. Primary antibodies (see antibody section) were diluted in PBS plus 3% BSA and incubated for 1 h. Cells were washed three times with PBS with the secondary antibody (see antibody section) being diluted into PBS plus 3% BSA and incubated for 1 h. Cells were washed three times with PBS and washed once with distilled water before mounting the coverslips in Fluoromount-G (Thermo Fisher).

### Microscopy and image analysis

For image acquisition, a Leica SP5-AOBS confocal laser scanning confocal microscope was used attached to a Leica DM I6000 inverted epifluorescence microscope. A 63× HCX PL APO oil lens and standard acquisition software and detectors were used. Once acquired, Pearson's correlation colocalisation and signal intensity analyses were quantified using Volocity 6.3 software (PerkinElmer). Image and line scan analysis was completed using ImageJ FIJI software. GraphPad Prism 7 was used for presentation and statistical analysis of data.

Live-cell imaging was performed using a Leica SP8 AOBS confocal laser scanning microscope attached to a Leica DM I6000 inverted epifluorescence microscope. The adaptive focus control was used to prevent drift of the *Z-*plane over time. The two hybrid GaAsP detectors were used to enable low laser settings. Images were acquired using the 63× HC PL APO CS2 lens and a speed of one image per 10 s. Imaging was performed at 37°C and 2× rapalog DMEM complete media was added to the pre-selected cell.

## Supplementary Material

Supplementary information
