# Supplementary material for: Acute inactivation of retromer and ESCPE-1 leads to time-resolved defects in endosomal cargo sorting
Source: J Cell Sci. 2020 Aug 3;133(15):jcs246033. doi: 10.1242/jcs.246033 (PMC7420817; doi:10.1242/jcs.246033)
Supplement: Supplementary information [file joces-133-246033-s1.pdf]

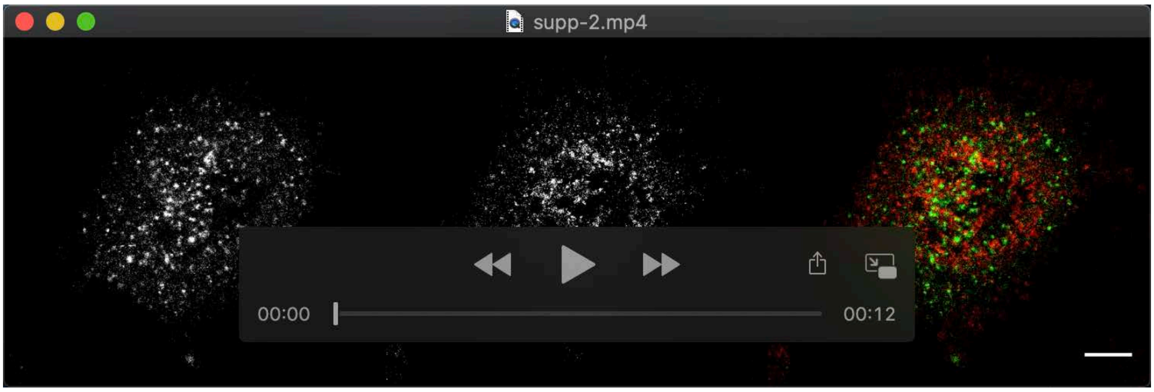

Movie 1A

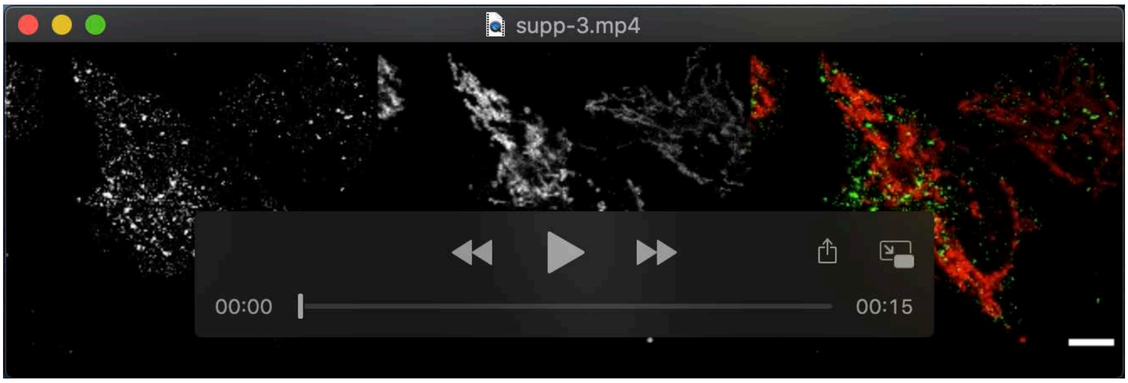

Movie 1B

Movies – **Temporal resolution of retromer knocksideways onto acceptor compartments after the addition of rapalog.** (A) Retromer (peroxisomal acceptor compartment) knocksideways HeLa cells were imaged with a frame being taken every 10 seconds after the addition of rapalog. Channels from left to right: 1) VPS35-GFP-FRB (Green), 2) PEX-RFP-FKBP (Red), 3) Merged. The merged panel shows both the VPS35-GFP-FRB and PEX-RFP-FKBP channels. Scale bars = 10 µm. Time display 00:00 min:sec. (B) Retromer (mitochondrial acceptor compartment) knocksideways HeLa cells were imaged with a frame being taken every 10 seconds after the addition of rapalog. Channels from left to right: 1) VPS35-GFP-FRB (Green), 2) Mito-RFP-FKBP (Red), 3) Merged. The merged panel shows both the VPS35-GFP-FRB and Mito-RFP-FKBP channels. Scale bars = 10 µm. Time display 00:00 min:sec.

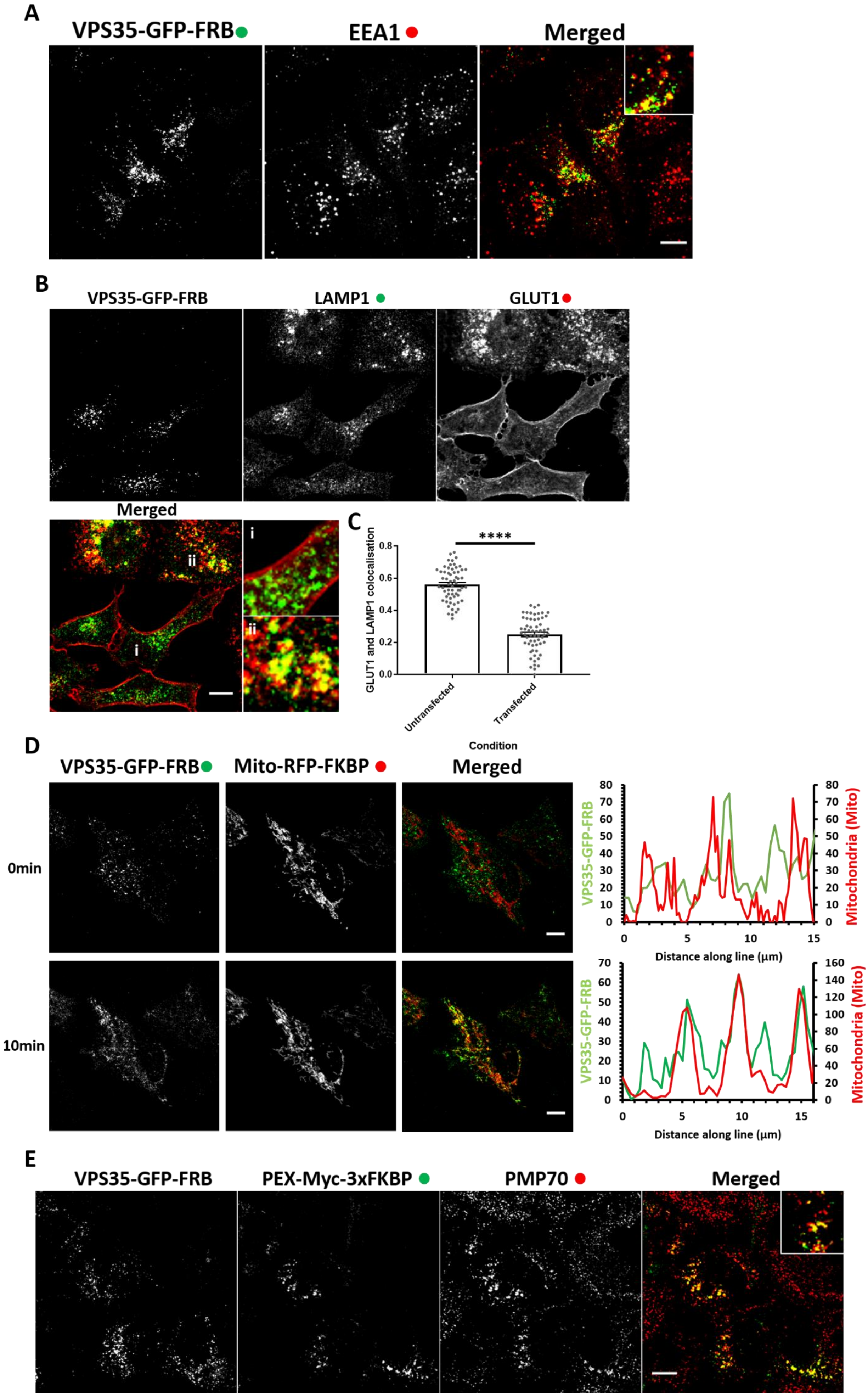
A
VPS35-GFP-FRB
EEA1
Merged
B
VPS35-GFP-FRB
LAMP1
GLUT1
Merged
i
ii
C
****
GLUT1 and LAMP1 colocalisation
0.8
0.6
0.4
0.2
0.0
Untransfected
Transfected
Condition
D
VPS35-GFP-FRB
Mito-RFP-FKBP
Merged
0min
10min
VPS35-GFP-FRB
Mitochondria (Mito)
Distance along line (µm)
E
VPS35-GFP-FRB
PEX-Myc-3xFKBP
PMP70
Merged

Figure S1 – **Retromer knocksideways is dynamic and is functionally active.** (A) VPS35 knocksideways cells were fixed and then labelled with anti-EEA1. Both the VPS35-GFP-FRB and EEA1 channels are shown in the merged panel with a zoom in panel. Scale bars = 10 µm (B) VPS35 knockout cells were transfected with retromer knocksideways and then fixed and labelled with anti-LAMP1 and anti-GLUT1. The merged panel shows both LAMP1 and GLUT1 channels and two zoom-in panels are displayed showing a VPS35-GFP-FRB expressing cell (i) and a cell not expressing retromer (ii, VPS35 knockout cell). Scale bars = 10 µm. (C) Pearsons co-localization between LAMP1 and GLUT1 in untransfected and transfected cells, $n_{\mathrm{exp}} = 3$, $n_{\mathrm{cell}} = 60$ with all data points being displayed. Statistical analysis performed – Welch's t-test ****<0.0001. (D) HeLa cells transfected with retromer knocksideways (VPS35-GFP-FRB and Mito-RFP-FKBP). Still frames are shown from a movie (Supplementary Movies 1B) at either 0 minutes or 10 minutes after the addition of rapalog. Line scans were generated using ImageJ drawing a line through mitochondrial structures and represent the colocalization between VPS35-GFP-FRB and Mito-RFP-FKBP at each timepoint. The merged panel displays both channels. Scale bars = 10 µm. (E) HeLa cells were transfected with retromer knocksideways (Peroxisomal, PEX-myc-3xFKBP acceptor compartment) and then antibody labelled with anti-myc and the peroxisomal marker anti-PMP70. The merged panel displays both PEX-myc-3xFKBP and PMP70. Scale bars = 10 µm.

Journal of Cell Science • Supplementary information

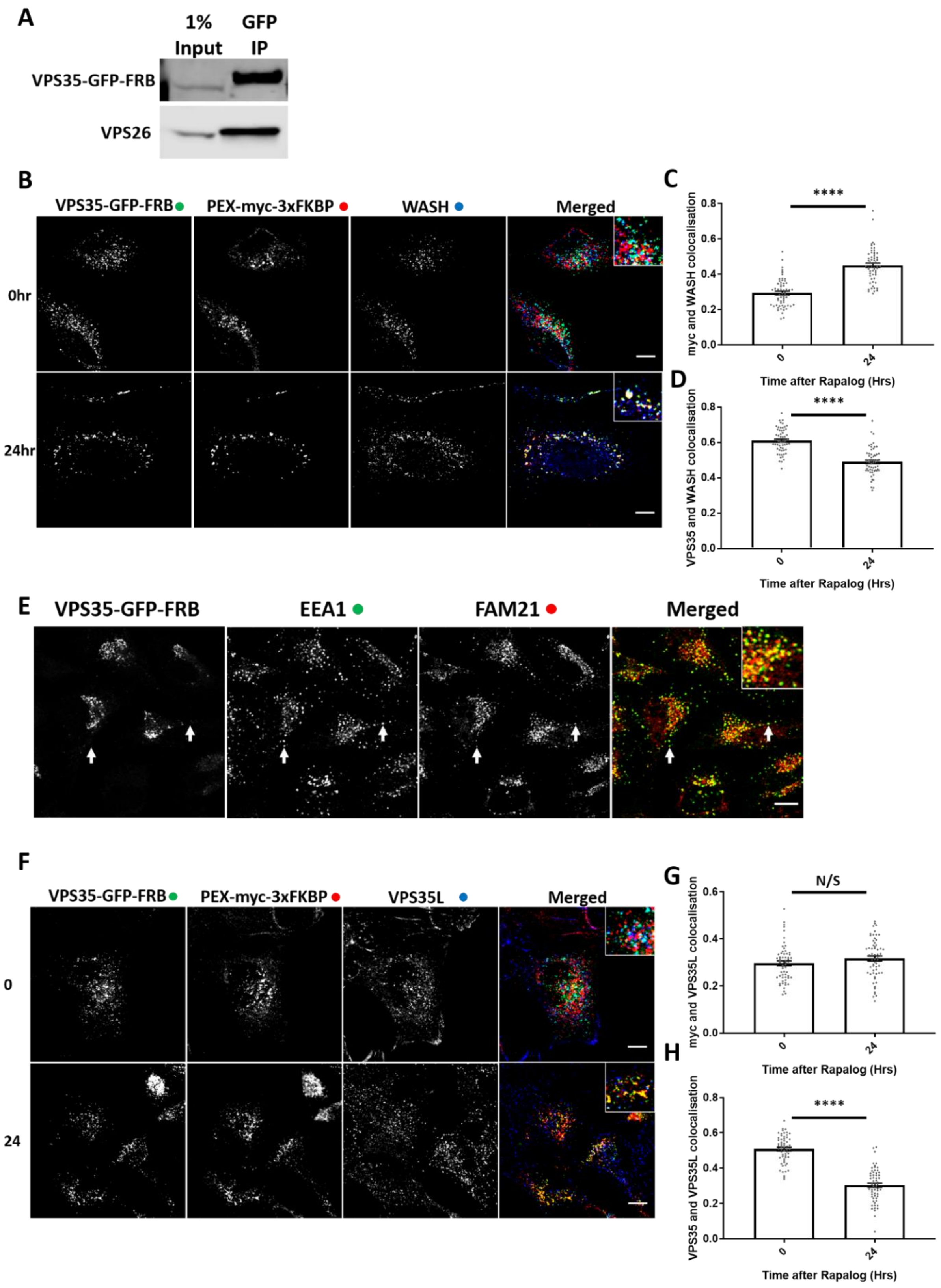

A
1% Input
GFP IP
VPS35-GFP-FRB
VPS26
B
VPS35-GFP-FRB
PEX-myc-3xFKBP
WASH
Merged
0hr
24hr
C
myc and WASH colocalisation
****
Time after Rapalog (Hrs)
D
VPS35 and WASH colocalisation
****
Time after Rapalog (Hrs)
E
VPS35-GFP-FRB
EEA1
FAM21
Merged
F
VPS35-GFP-FRB
PEX-myc-3xFKBP
VPS35L
Merged
0
24
G
myc and VPS35L colocalisation
N/S
Time after Rapalog (Hrs)
H
VPS35 and VPS35L colocalisation
****
Time after Rapalog (Hrs)

Figure S2 – **Retromer knocksideways used as an *in cellulo* interaction assay.** (A) Western blot analysis of a GFP immunoprecipitation experiment using VPS35-GFP-FRB transiently expressed in HEK293T cells and blotting for VPS35 and VPS26. Both the input and the immunoprecipitation samples are shown. (B) Retromer knocksideways HeLa cells were fixed before or after 24 hours of rapalog addition and then labelled with anti-myc and anti-WASH. The merged panel displays all three channels with zoom-in panels. Scale bars = 10 µm. (C) Pearsons colocalization between myc and WASH before and after 24 hours of rapalog, n_{exp} = 3, n_{cell} = 60 with all data points being displayed. Statistical analysis performed – Welch's t-test, ****<0.0001. (D) Pearsons co-localization between VPS35-GFP-FRB and WASH before and after 24 hours of rapalog, n_{exp} = 3, n_{cell} = 60 with all data points being displayed. Statistical analysis performed Welch's t-test ****<0.0001. (E) Retromer knocksideways HeLa were fixed after 24 hours of rapalog addition and stained using anti-EEA1 and anti-FAM21. The merged panel displays both the EEA1 and FAM21 channels with a zoom in panel. Scale bars = 10 µm. Arrows on each panel display areas of co-localization between EEA1 and FAM21. (F) Retromer knocksideways HeLa cells were fixed before or after 24 hours of rapalog addition and then labelled with anti-myc and anti-VPS35L. The merged panel displays all three channels with zoom-in panels. Scale bars = 10 µm. (G) Pearsons co-localization between myc and VPS35L before and after 24 hours of rapalog, n_{exp} = 3, n_{cell} = 60 with all data points being displayed. Statistical analysis performed - Welch's t-test, N/S >0.05. (H) Pearsons colocalization between VPS35-GFP-FRB and VPS35L before and after 24 hours of rapalog, n_{exp} = 3, n_{cell} = 60 with all data points being displayed. Statistical analysis performed – Welch's t-test, ****<0.0001.

Journal of Cell Science • Supplementary information

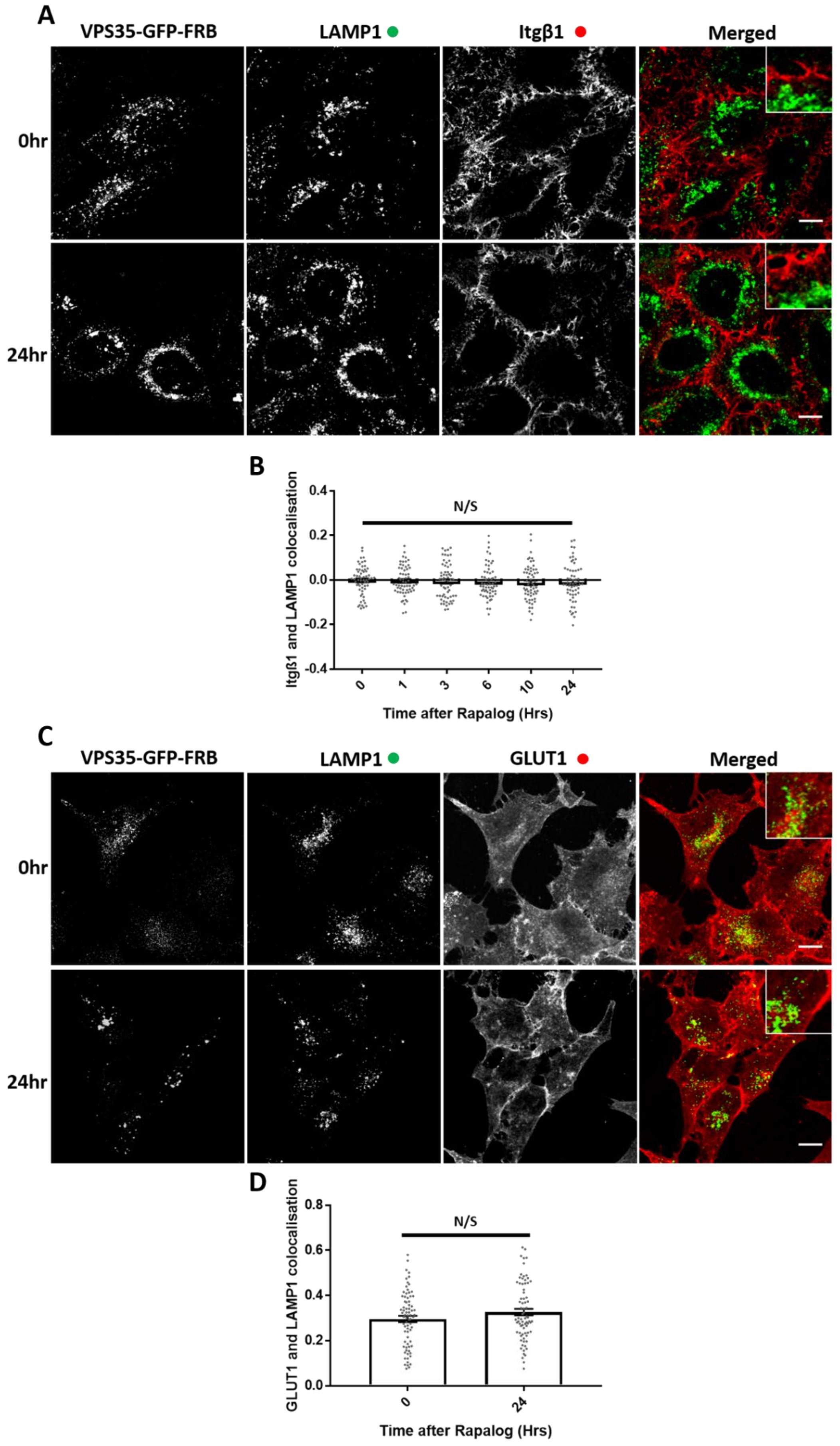
A
VPS35-GFP-FRB
LAMP1
Itgβ1
Merged
0hr
24hr
B
Itgβ1 and LAMP1 colocalisation
N/S
0.4
0.2
0.0
-0.2
-0.4
0
1
3
6
10
24
Time after Rapalog (Hrs)
C
VPS35-GFP-FRB
LAMP1
GLUT1
Merged
0hr
24hr
D
GLUT1 and LAMP1 colocalisation
N/S
0.8
0.6
0.4
0.2
0.0
0
24
Time after Rapalog (Hrs)

Figure S3 – **Retromer knocksideways specifically inactivates retromer recycling.** (A) Retromer knocksideways HeLa cells were fixed before and after the addition of rapalog for multiple timepoints and then labelled for anti-LAMP1 and anti-Itgβ1. The merged panel shows both the LAMP1 and Itgβ1 channels with a zoom panel. Scale bars = 10 µm. (B) Pearsons co-localization between LAMP1 and Itgβ1 before and after multiple timepoints of rapalog addition, n_{exp} = 3, n_{cell} = 60 with all data points being displayed. Statistical analysis performed - Ordinary one-way ANOVA with multiple comparisons, N/S >0.05. (C) Retromer knocksideways was transfected into wild-type HeLa cells and were fixed before and after the addition of rapalog for 24 hours and then labelled for anti-LAMP1 and anti-GLUT1. The merged panel shows both the LAMP1 and GLUT1 channels with a zoom panel. Scale bars = 10 µm. (D) Pearsons co-localization between LAMP1 and GLUT1 before and after 24 hours of rapalog, n_{exp} = 3, n_{cell} = 60 with all data points being displayed. Statistical analysis performed – Welch’s t-test, N/S >0.05.

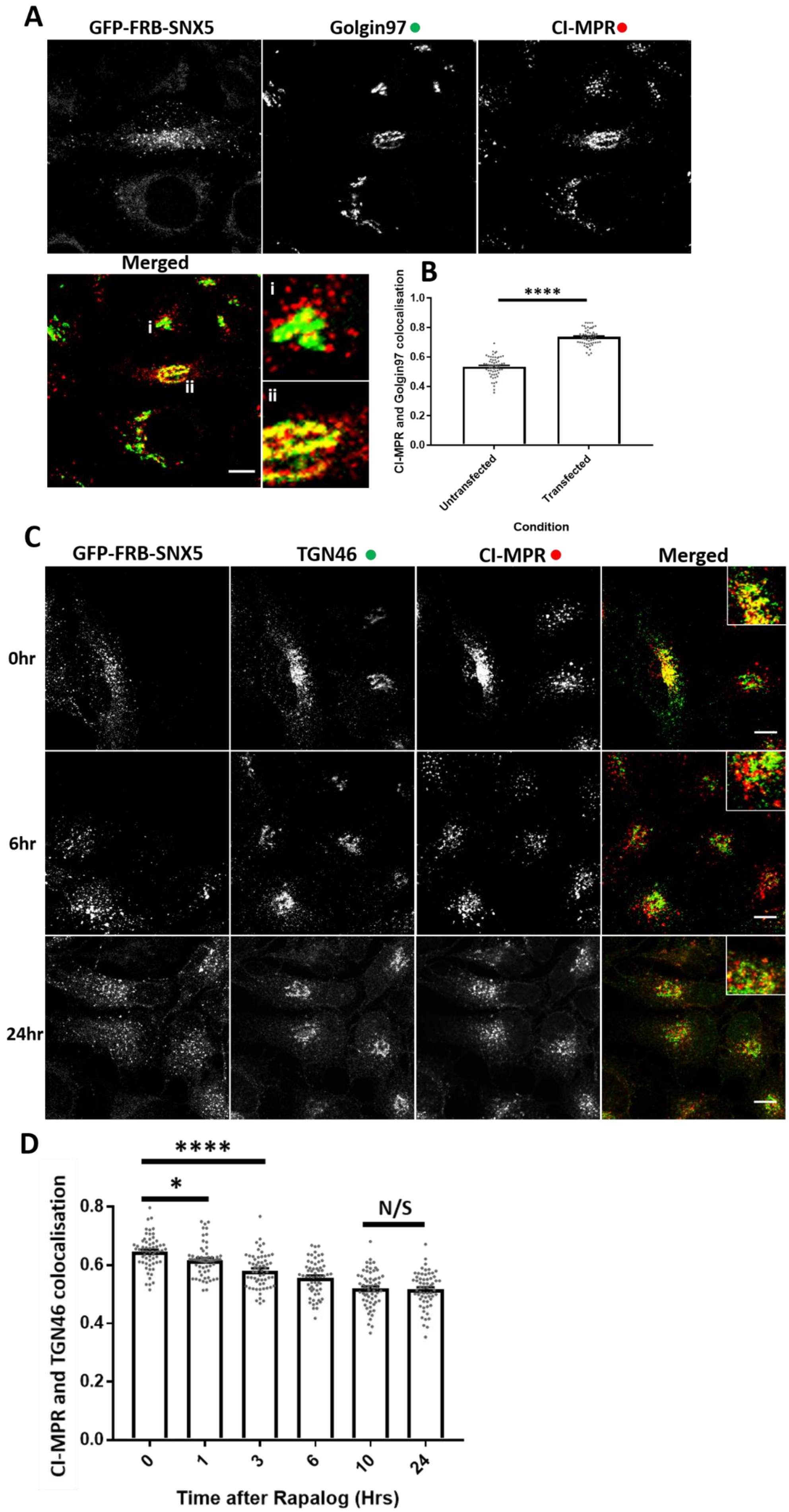

Journal of Cell Science • Supplementary information

Figure S4 – **ESCPE-1 knocksideways rescues SNX5/SNX6 dual knockout cells phenotypes and allows the time-resolved redistribution of CI-MPR redistribution after rapalog.** (A) Dual SNX5/SNX6 knockout cells were transfected with SNX-BAR knocksideways and fixed and labelled with anti-Golgin97 and anti-CI-MPR. The merged panel shows Golgin97 and CI-MPR channels and two zooms showing a cell not expressing GFP-FRB-SNX5 (i, SNX5/SNX6 knockout cell) and a transfected GFP-FRB-SNX5 expressing cell (ii). Scale bars = 10 µm. (B) Pearsons co-localization between Golgin97 and CI-MPR in untransfected and transfected cells, n_{exp} = 3, n_{cell} = 60 with all data points being displayed. Statistical analysis performed – Welch's t-test, ****<0.0001. (C) SNX-BAR knocksideways HeLa cells were fixed before and after the addition of rapalog and labelled for anti-TGN46 and anti-CI-MPR. The merged panel shows both the TGN46 and CI-MPR channels with a zoom panel. Scale bars = 10 µm. (D) Pearsons co-localization between TGN46 and CI-MPR before and after multiple timepoints of rapalog, n_{exp} = 3, n_{cell} = 60 with all data points being displayed. Statistical analysis performed - Ordinary one-way ANOVA with multiple comparisons, ****<0.0001, *<0.05, N/S >0.05.

Journal of Cell Science • Supplementary information

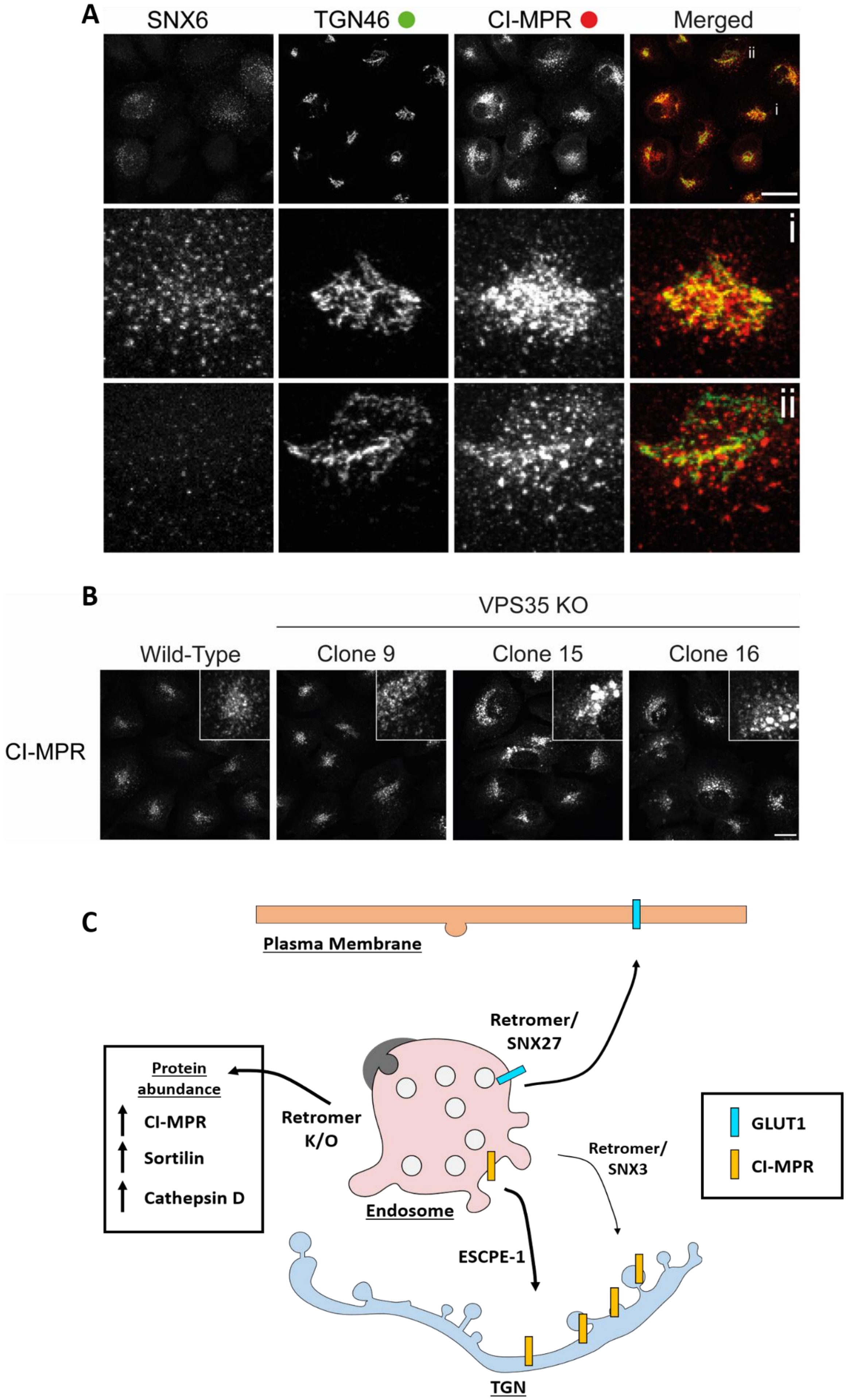

Journal of Cell Science • Supplementary information

Figure S5 – **CI-MPR expression is upregulated in VPS35 knockout H4 cells and redistributed away from the *trans*-Golgi network in SNX5/SNX6 knockout H4 cells.** (A) Dual SNX5/SNX6 knockout H4 neuroblastoma cells mixed population were fixed and stained with anti-CI-MPR, anti-TGN46 and anti-SNX6. The merged panel shows both TGN46 and CI-MPR and two zooms showing a (i) SNX5/SNX6 positive cell and a (ii) SNX5/SNX6 knockout cell. Scale bars = 20 µm. (B) VPS35 knockout clonal cells fixed and stained with anti-CI-MPR. Scale bars = 20 µm. (C) Cartoon model depiction of the cellular roles of Retromer and ESCPE-1 investigated in this study.

Journal of Cell Science • Supplementary information
